# A mixing heteroduplex mobility assay (mHMA) to genotype homozygous mutants with small indels generated by CRISPR-Cas9 nucleases

**DOI:** 10.1016/j.mex.2018.11.017

**Published:** 2018-11-27

**Authors:** Samantha D. Foster, Sarah R. Glover, Ashley N. Turner, Kiranam Chatti, Anil K. Challa

**Affiliations:** a Science and Technology Honors Program, University of Alabama at Birmingham (UAB), United States of America; b Undergraduate Neuroscience Program, UAB, United States of America; c Department of Biology, UAB, United States of America; d Dr. Reddy’s Institute of Life Sciences, Hyderabad, India; e Department of Genetics, UAB, United States of America

**Keywords:** Mixing heteroduplex mobility assay (mHMA) for screening homozygous mutants, HMA, Homozygosity, Genotyping, PCR, Gene editing, Zebrafish, Simple CRISPR-Cas9

## Abstract

Abstract

The development of gene editing technologies, especially the CRISPR-Cas9 system, has been pivotal for understanding the functional role of proteins. Rapid and efficient genotyping methods are necessary to screen for generated mutations and streamline the isolation of homozygotes. CRISPR-Cas9 system targeting a single site in the gene typically results in small indels. Many genotyping methods utilize the heteroduplex that is formed when wild-type and mutant amplicons with small indels anneal during PCR creating a bubble due to mismatched strands. These methods include T7 endonuclease/Cel-I assay, high resolution melting (HRM) analysis, and heteroduplex mobility assay (HMA).

Our protocol explains a simple, two step method of a mixing HMA (mHMA) to identify homozygous mutants, a modification of the previously published HMA. We have utilized the mHMA for screening and genotyping numerous CRISPR generated models.

The mHMA method to differentiate homozygous wild type from homozygous mutant animals eliminates -

• DNA sequencing, even with small indels that can be difficult to discern on a gel.
• additional enzymatic reaction steps, such as with the T7EI/Cel-I assay.
• specialized equipment and analysis tools, such as with HRM analysis.

**Figure:**
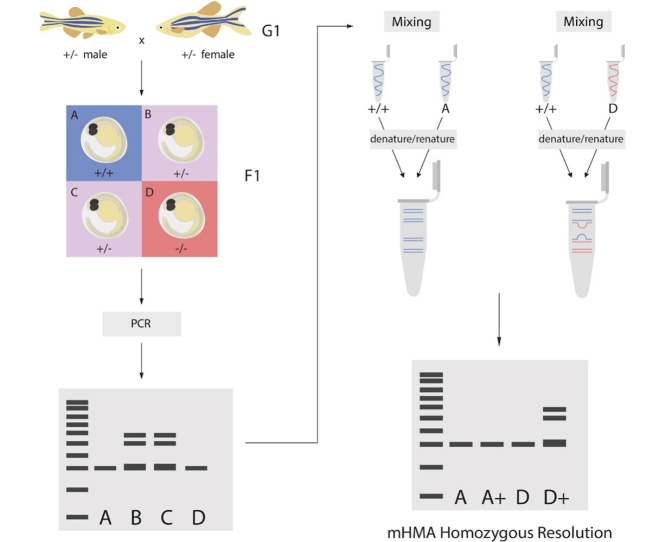

Specifications table

**Table:** 

Subject Area	Biochemistry, Genetics and Molecular Biology
More specific subject area:	Genotyping method for CRISPR-Cas9 generated homozygous mutations
Method name:	Mixing heteroduplex mobility assay (mHMA) for screening homozygous mutants
Name and reference of original method	S. Ota, Y. Hisano, M. Muraki, K. Hoshijima, T.J. Dahlem, D.J. Grunwald, Y. Okada, A. Kawahara. Efficient identification of TALEN-mediated genome modifications using heteroduplex mobility assays. *Genes to Cells* (2013) 18 (6):450–8.
Resource availability	N/A

## Method details

### Materials

• Polymerase chain reaction (PCR) tubes (strip format), 0.2mL
• Designed primer set for CRISPR target site (forward and reverse primers)
• *Taq* 2X Master Mix (M0270 L; New England Biology, Ipswich, MA)
• Nuclease-free water
• Thermocycler system
• Micropipettes
• Polyacrylamide gels (precast or handcast); reagents below if hand casting:
  ○ 5X tris-borate-EDTA (TBE) buffer
  ○ 40% acrylamide
  ○ 10% ammonium persulfate solution (APS)
  ○ Tetramethylethylenediamine (TEMED)
• Hand casting system (if making handcast gels)
• Electrophoresis chamber and power supply
• 10X TBE running buffer
• DNA loading dye
• DNA ladder (100 bp DNA ladder)
• DNA stain (ethidium bromide or alternative)
• Gel imaging system (ultraviolet (UV) imaging)
  • Note: Chemicals and other components can be used from any reliable company.

### Animal use

No animals were directly used in this study. However, genomic DNA samples used in this study were made available from animals that were generated under an approved Institutional Animal Care and Use Committee (IACUC) protocol at the University of Alabama at Birmingham.

### Abbreviations/generation nomenclature

G0- mutagenized generation (e.g. animals obtained from embryos injected with CRISPR-Cas9 nucleases)

G1- first generation following mutagenesis (G0 x wild type)

F1- first filial or inbreeding generation

## Procedure

A Genotyping modified organisms (F1) from a heterozygous (G1) cross using PCR and heteroduplex mobility assay (HMA)
  1 Acquire tissue biopsy (tail fin clip or embryo) and extract DNA using an alkali lysis and neutralization procedure. Use 50 ng of extracted genomic DNA as template to amplify a region flanking each CRISPR target site.
    a Note: Primer design and optimization of genotyping assay for each CRISPR target site is performed with wild-type DNA template prior to screening any modified organism, including G0 (i.e., gradient PCR for selection of optimum annealing temperature for a given primer set).
  2 Set up the following PCR for each individual F1 organism using NEB Taq 2x Master Mix and the optimized annealing temperature for the designed primer set:
    a PCR components - 12.5 μL volume PCR :
      i Taq 2x Master Mi × 6.25 μL
      ii Forward primer (20 μM) 0.25 μL
      iii Reverse primer (20 μM) 0.25 μL
      iv Nuclease-free water 4.75 μL
      v Template DNA (50 ng/uL)1.00 μL
    b Note: Include at least one heterozygous G1 parent as a positive control.
  3 Next, subject the amplicons to denaturation followed by a slow renaturation using a thermocycler (95℃ for 2 min, ramp down to 25℃ at 0.1℃ per second). Alternatively, a boiling water bath set to cool down on the benchtop can also be used for denaturation and slow renaturation of the amplicons. This facilitates the formation of heteroduplexes.
  4 Assemble 6–8% TBE polyacrylamide gels (precast or handcast) into electrophoresis chamber with 1X TBE running buffer.
  5 Mi x 1 μL DNA loading dye with 1–2 μL of each denatured/renatured PCR sample and load into individual wells of a polyacrylamide gel.
    a Note: Remember to save a well/lane for loading the DNA ladder, a heterozygous G1 parent positive control, a wild-type positive control, and no template control (NTC).
    b Note: Do not discard the remaining denatured/renatured PCR sample, it will be utilized in the next phase of mixing HMA.
  6 Ensure the electrophoresis chamber is filled with 1X TBE running buffer.
  7 Plug the electrophoresis chamber into a power supply and run gels at 150 V for 45–60 minutes.
    a Note: A good indicator for stopping the run is when the loading dye has completely run off the polyacrylamide gel.
  8 Remove polyacrylamide gels from electrophoresis chamber and plates and perform 5–10 minute post-stain with ethidium bromide or alternative.
  9 Image polyacrylamide gel with a UV imaging system.
  10 By this method of heteroduplex mobility assay (HMA), it is easy to identify heterozygous organisms. The heterozygous organism results in heteroduplex formation (upper and/or lower bands), whereas the homozygous wild-type and homozygous mutant organism result in homoduplex formation (single band).
    a Note: With small indels it can be difficult to identify a homozygous mutant individual, since it can be indistinguishable from the wild-type amplicon.
B Identification of homozygous mutant organisms (F1) using mixing HMA (mHMA)
  1 Identify all F1 organisms from the initial HMA experiment (A) that result in a single PCR band. Obtain their denatured/renatured PCR samples.
    a Note: These organisms are homozygous, but could be homozygous wild-type or homozygous mutant.
  2 Mix 1:1 ratio of the following in a new PCR tube:
    a PCR amplicon of a known wild-type organism (wild-type positive control from above if included)
    b PCR amplicon of the identified homozygous F1 organism resulting in a single PCR band
      i Note: There will be sufficient volume from the initial PCR to carry out this mixing experiment (so, 2 μL:2 μL).
      ii Note: Depending on the number of homozygous organisms being screened, additional PCR amplicon of a known wild-type organism might need to be prepared.
  3 Next, subject this PCR amplicon mixture to denaturation followed by a slow renaturation process using a thermocycler (same process as above).
  4 Resolve these mixed samples on 6–8% polyacrylamide gels (same process as above).
  5 Compare results from first round and identify organisms whose banding patterns change from a single band to a mobility profile that matches the heterozygous parent.
    a Note: The original denatured/renatured PCR amplicon of each identified F1 homozygous organism can be loaded alongside the corresponding PCR mixture for visualization on the same gel.
  6 By this method of mHMA, it is easy to identify homozygous mutant from homozygous wild type. By mixing PCR amplicon of a known wild type with that of an identified homozygous organism, this forces heteroduplexes to form if the identified homozygous organism is a homozygous mutant. A homozygous wild-type organism will still show a single band (homoduplexes) after this mHMA experiment.

## Method validation

In CRISPR experiments, we use the HMA for identifying mutations in G0 organisms and genotyping heterozygous organisms in G1, and both heterozygous and homozygous organisms in F1 and subsequent breedings. Optimization of the primer set and genotyping assay are key to a successful screening strategy with HMA. The mHMA can be used to differentiate homozygous null mutations from wild type in a relatively simple and inexpensive method using HMA. Homozygosity of the null mutation is confirmed using visualization via gel electrophoresis. The mHMA is validated in our studies with several CRISPR-Cas9 generated model systems, in addition to zebrafish.

**Fig. 1:**
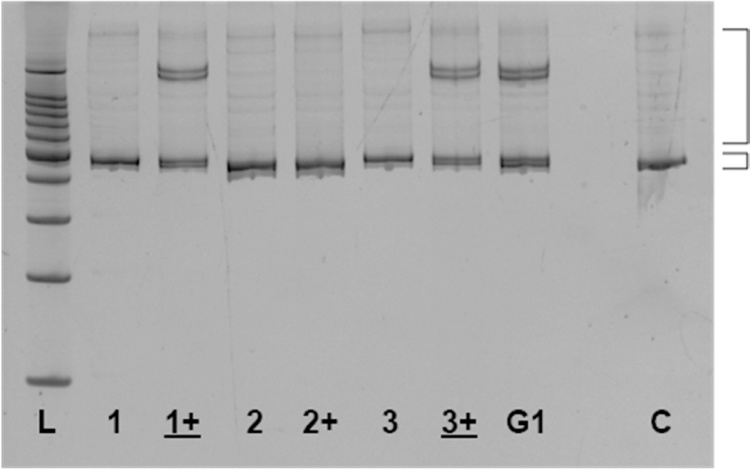
**CRISPR homozygous null mutation detection by mixing heteroduplex mobility assay (mHMA) in zebrafish.** Image of ethidium bromide stained polyacrylamide gel (6%) showing separation of homoduplex and heteroduplex PCR amplicons. Amplicons were obtained from three HMA-identified homozygous F1 zebrafish generated from a G1 heterozygous cross. Small and large brackets indicate homoduplex and heteroduplex bands respectively. The initial F1 HMA PCR showing a single band (1–3) is loaded first with mixed WT/F1 PCR (1+, 2+, 3+) loaded in the next lane to the right for direct comparison. Underlined # + = inferred homozygous mutant; Not underlined # + = inferred homozygous wild type; L = 100 bp ladder; G1 = G1 heterozygous parent; C = wild-type control.

As discussed, while heterozygotes carrying small indels are unambiguously identified using HMA, homozygous mutants with the same small indels can be difficult to identify using HMA; PCR amplicons from these homozygous null alleles migrate very close to the wildtype amplicons and are indistinguishable from the wild-type amplicon. Homozygosity of a small indel mutation can be confirmed using mHMA by gel visualization as shown in Fig. 1. Mixing the PCR amplicon of a known wild-type organism with that of identified homozygous organisms (1:1) takes advantage of heteroduplex formation, forcing the mobility profile of a homozygous mutant organism to match that of a heterozygous organism after mixing.

## Additional information

The development of new gene editing technologies has been important for understanding the functional role of proteins and ultimately elucidating the complexity of organisms. Current gene editing technologies can be used to generate mutations via non-homologous end joining (NHEJ), creating null alleles and non-functional proteins. The consequences resulting from homozygous loss-of-function mutations can be analyzed in model organisms such as zebrafish to understand protein structure and function.

To analyze protein function, complete knockout organisms with homozygous mutations must be generated. The mutated generation (from technologies such as CRISPR/Cas9, TALENs, and zinc finger nucleases) are known to be mosaics because each founder may contain several mutations depending the persistence and activity of the nuclease during subsequent cell divisions. As the cell repairs the double stranded breaks created with these nucleases. they may undergo either NHEJ or homology-directed repair (HDR). Founder organisms can be identified by PCR using tissue biopsy, which can harbor multiple indels in a given G0 founder. Since not all mutations in the G0 organism will transmit through the germline, it is best to screen the G1 offspring from outcrossing to a wild type for germline transmission and analyze the frequency of unique mutations. Once identified, a G1 sibling pair with the identical mutation can be inbred to generate the homozygous mutant organism for functional studies. The expected Mendelian distribution of 1:2:1 of heterozygous and homozygous F1 organisms requires additional screening.

Rapid and efficient genotyping methods are necessary to screen for relevant mutations and streamline the isolation of mutations to homozygosity. Many genotyping methods utilize the heteroduplex that is formed when wild-type and mutant amplicons anneal during PCR, creating a bubble due to the mismatched strands. Some of the methods available to genotype heterozygous organisms using heteroduplexes include T7 endonuclease/Cel-I assay, high resolution melting (HRM) analysis, and heteroduplex mobility assay (HMA).

The HMA is based on the differential mobility of DNA molecules with and without mismatches and provides an easy and convenient method to discern heterozygous organisms prior to confirmation by sequencing. This method requires polyacrylamide gel electrophoresis equipment to differentiate G1 individuals, while the T7EI/Cel-I assay requires additional reaction steps and HRM method requires specialized equipment and software. The mHMA is an extension of HMA, and can be used to differentiate homozygous null mutations from wild type in a relatively simple way that can be performed with instruments readily available in a molecular biology laboratory [1].

## Conflict of interest

The authors declare that there are no conflicts of interest.

## References

[1] Ota S., Hisano Y., Muraki M., Hoshijima K., Dahlem T.J., Grunwald D.J., Okada Y., Kawahara A. (2013). Efficient identification of TALEN-mediated genome modifications using heteroduplex mobility assays. Genes Cells.

